# 1,5-anhydro-D-fructose induces anti-aging effects on aging-associated brain diseases by increasing 5’-adenosine monophosphate-activated protein kinase activity via the peroxisome proliferator-activated receptor-γ co-activator-1α/brain-derived neurotrophic factor pathway

**DOI:** 10.18632/aging.205228

**Published:** 2023-11-09

**Authors:** Kiyoshi Kikuchi, Shotaro Otsuka, Seiya Takada, Kazuki Nakanishi, Kentaro Setoyama, Harutoshi Sakakima, Eiichiro Tanaka, Ikuro Maruyama

**Affiliations:** 1 Division of Brain Science, Department of Physiology, Kurume University School of Medicine, Fukuoka 830-0011, Japan; 2 Department of Neurosurgery, Kurume University School of Medicine, Fukuoka 830-0011, Japan; 3 Department of Systems Biology in Thromboregulation, Kagoshima University Graduate School of Medical and Dental Sciences, Kagoshima 890-8520, Japan; 4 Course of Physical Therapy, School of Health Sciences, Faculty of Medicine, Kagoshima University, Kagoshima 890-8544, Japan; 5 Division of Laboratory Animal Resources and Research, Center for Advanced Science Research and Promotion, Kagoshima University, Kagoshima 890-8520, Japan

**Keywords:** AMP-activated protein kinases, brain-derived neurotrophic factor, peroxisome proliferator-activated receptors, blood pressure, aging

## Abstract

5’-Adenosine monophosphate-activated protein kinase (AMPK) is a metabolic sensor that serves as a cellular housekeeper; it also controls energy homeostasis and stress resistance. Thus, correct regulation of this factor can enhance health and survival. AMPK signaling may have a critical role in aging-associated brain diseases. Some *in vitro* studies have shown that 1,5-anhydro-D-fructose (1,5-AF) induces AMPK activation. In the present study, we experimentally evaluated the effects of 1,5-AF on aging-associated brain diseases *in vivo* using an animal model of acute ischemic stroke (AIS), stroke-prone spontaneously hypertensive rats (SHRSPs), and the spontaneous senescence-accelerated mouse-prone 8 (SAMP8) model. In the AIS model, intraperitoneal injection of 1,5-AF reduced cerebral infarct volume, neurological deficits, and mortality. In SHRSPs, oral administration of 1,5-AF reduced blood pressure and prolonged survival. In the SAMP8 model, oral administration of 1,5-AF alleviated aging-related decline in motor cognitive function. Although aging reduced the expression levels of peroxisome proliferator-activated receptor-γ co-activator-1α (PGC-1α) and brain-derived neurotrophic factor (BDNF), we found that 1,5-AF activated AMPK, which led to upregulation of the PGC-1α/BDNF pathway. Our results suggest that 1,5-AF can induce endogenous neurovascular protection, potentially preventing aging-associated brain diseases. Clinical studies are needed to determine whether 1,5-AF can prevent aging-associated brain diseases.

## INTRODUCTION

5′-Adenosine monophosphate-activated protein kinase (AMPK) is an evolutionarily conserved serine/threonine kinase that is crucial for the maintenance of cellular energy homeostasis; various molecular mechanisms and physiological processes regulate AMPK activity. At cellular and systemic levels, AMPK controls critical determinants of aging and longevity. AMPK regulates various physiological and metabolic processes; it is dysregulated in major chronic diseases.

Brain aging research has been an important research focus in recent years. While the brain clearly changes with increasing age, there is less clarity concerning the rate of change, biological age of the brain, and processes involved. Cognition and behavior are affected by aging-related changes at multiple levels, from single molecules to whole organs. Incidences of stroke, white matter lesions, and dementia increase with age, as does the level of memory impairment [1]. AMPK regulation has been implicated in the pathophysiology of brain aging, including conditions such as stroke and Alzheimer’s disease (AD) [2, 3]. Activation of AMPK results in reduced blood pressure (BP) [4], enhanced fatty acid oxidation, and decreased levels of glucose and lipids (e.g., cholesterol and triglycerides) [5]. High expression of AMPK in brain tissue is linked to its ability to protect against ischemic stimuli and promote autophagy [6]; the activation of AMPK may protect against ischemic stroke [7]. Moreover, AMPK can promote neurological improvement and neurogenesis, attenuate cerebral hemorrhage, and alleviate some neurodegeneration [7]. However, few studies have clarified the usefulness of AMPK in clinical practice; its mechanisms are unclear. Future clinical studies of AMPK may help to prevent aging-associated brain diseases.

Aging-related pathways (e.g., AMPK) are major targets of anti-aging interventions. Exercise and metformin are indirect AMPK activators, and metformin reportedly extends longevity in *Caenorhabditis elegans* through a dietary restriction-like mechanism via AMPK [8]. Furthermore, exercise [9] and metformin [10] exhibit neuroprotective effects in animal models of aging-associated brain diseases; they are currently the focus of clinical trials to determine their effects on human aging, particularly in terms of tissue homeostasis and metabolic dysfunction.

1,5-Anhydro-D-fructose (1,5-AF) induces the activation of AMPK. 1,5-AF is a bioactive monosaccharide produced by the degradation of starch and glycogen [11]. It is present in mammalian tissues such as rat liver, and is metabolized *in vivo* to 1,5-anhydro-D-glucitol. 1,5-AF has a wide spectrum of bioactive properties, including antioxidant, anti-inflammatory, antimicrobial, antidiabetic, and anticancer effects. We recently found that 1,5-AF activates AMPK in PC12 neuron-like cells *in vitro* [12]. Because 1,5-AF activates AMPK, we hypothesized that the administration of 1,5-AF would prevent aging-associated brain diseases. To our knowledge, there have been no reports concerning the effects of 1,5-AF on aging-associated brain diseases *in vivo*; thus, we investigated these effects in multiple animal models.

Because brain aging in humans is heterogeneous and has a complex pathophysiology, it is impossible to mimic all aspects in a single animal model. The 10 most common causes of death worldwide include two diseases related to brain aging: stroke and dementia (AD and other dementias). Accordingly, we used animal models of stroke and dementia.

Stroke-prone spontaneously hypertensive rats (SHRSPs) have severe hypertension and a high risk of aging-related stroke [13]; most SHRSPs die of stroke. These rats are widely used as a model for human stroke; they are suitable for both mechanistic and treatment studies [14]. In the SHRSP Izumo strain (SHRSP/Izm; isolated from Wistar Kyoto rats (WKY/Izm) [14]), BP begins increasing shortly after birth; it reaches 250 mmHg by 18 weeks of age and 300 mmHg by 20 weeks of age [15]. In contrast, BP in WKY/Izm rats remains at 140–150 mmHg from 6 to 30 weeks of age [15]. Most SHRSP/Izm rats die of hemorrhagic or ischemic stroke [14].

The spontaneous senescence-accelerated mouse-prone (SAMP) model is widely used. SAMP and senescence-accelerated mouse-resistant mice were selected from AKR/J mice by the Takeda laboratory [16]; each SAMP strain exhibits specific aging-related disease phenotypes. Changes in the SAMP8 strain are generally similar to the pathomorphology in aging human brains; these mice exhibit several specific glioneuronal responses. Therefore, SAMP8 mice offer a model for accelerated senescence, as well as AD and other cognitive disorders [16]. SAMP8 mice exhibit most pathological features of AD, including abnormal expression of anti-aging factors; increased amyloid-β deposition, tau hyperphosphorylation, inflammation, oxidative stress, and endoplasmic reticulum stress, abnormal autophagy activity, and intestinal flora disruption [17]. Thus, SAMP8 mice enable effective visualization of AD and exploration of new therapeutic targets.

Because their pathomorphologies are similar to clinical findings in human patients, we used these animal models to inspect the effects of 1,5-AF on aging-associated brain diseases. Our primary objective was to validate 1,5-AF in multiple animal models for subsequent translation into clinical applications. We also examined the molecular mechanisms underlying the effects of 1,5-AF. Exercise-induced enhancement of AMPK activity and upregulation of the peroxisome proliferator-activated receptor-γ co-activator-1α (PGC-1α) and brain-derived neurotrophic factor (BDNF) pathway may contribute to the beneficial effects of exercise on amyloid-β-induced impairments of learning and memory [18]. PGC-1α is a downstream effector of AMPK [19], and BDNF is a downstream effector of PGC-1α [20].

Recently, we reported that 1,5-AF activates PGC-1α via AMPK, with potential mitochondrial biogenesis and cytoprotective effects in PC12 cells [12]. Mitochondria have been implicated in aging, as well as in the onset and progression of several aging-related diseases, for decades. Aging is a risk factor for various degenerative diseases; while aging and its related diseases have multiple causative factors, mitochondrial dysfunction is an important contributing factor and may be mediated by insufficient adenosine triphosphate supply [21]. PGC-1α is a key regulator of mitochondrial biogenesis [22]; this transcriptional co-activator helps to regulate the expression patterns of energy metabolism-related genes and contributes to oxidative phosphorylation and mitochondrial integrity [21].

Brain aging has been linked with neurotrophins, a class of growth/survival factors. BDNF is the most abundant neurotrophin in the central nervous system; it has diverse intrinsic regulatory mechanisms and functions [23, 24]. During aging-related synaptic loss, BDNF prevents cerebral atrophy and cognitive decline; it also interacts with reactive oxygen species that can exacerbate aging, neurodegenerative diseases, and some neuropsychiatric disorders [25]. Thus, we investigated the effects of 1,5-AF on the AMPK/PGC-1α/BDNF pathway in multiple animal models of human aging-associated brain diseases.

## RESULTS

### Intraperitoneal injection of 1,5-AF reduces cerebral infarct volume in rats with acute ischemic stroke (AIS)

**Figure 1:**
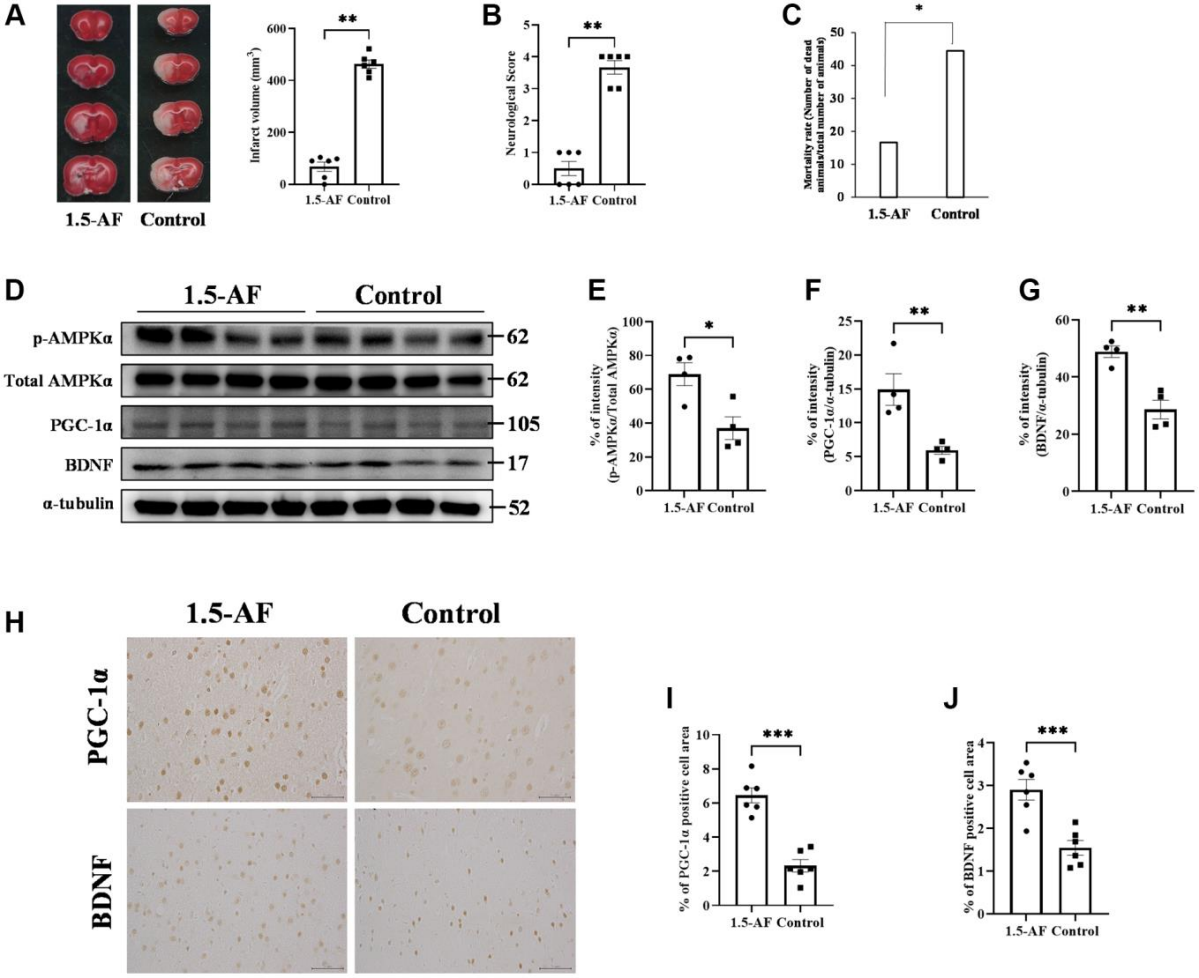
**1,5-AF activates pAMPK, decreases infarct volume, and reduces neurological deficits.** Representative TTC-stained cerebral sections from 1,5-AF and control rats (**A**); infarct volume (white region) was smaller in 1,5-AF rats than in control rats (1,5-AF: *n* = 6, control: *n* = 6). Neurological scores (1,5-AF: *n* = 6, control: *n* = 6) (**B**) and mortality rate (1,5-AF: *n* = 6, control: *n* = 6) (**C**) after AIS. Neurological scores were better and mortality rate was lower in 1,5-AF rats than in control rats. Immunoblotting results after AIS (1,5-AF: *n* = 4, control: *n* = 4) (**D**). Semiquantitative analysis of immunoblots revealed that protein levels of pAMPK (**E**), PGC-1α (**F**), and BDNF (**G**) were higher in 1,5-AF rats than in control rats. Photomicrographs of PGC-1α and BDNF immunoreactivities (1,5-AF: *n* = 6, control: *n* = 6) (**H**). PGC-1α (**I**) and BDNF (**J**) expression was higher in 1,5-AF rats than in control rats. Data are shown as the mean ± standard error. ^*^p < 0.05, ^**^p < 0.01, ^***^p < 0.001. Scale bar = 50 μm. Abbreviations: 1,5-AF: 1,5-anhydro-D-fructose; AIS: acute ischemic stroke; BDNF: brain-derived neurotrophic factor; pAMPK: phosphorylated 5’-adenosine monophosphate-activated protein kinase; PGC-1α: peroxisome proliferator-activated receptor-γ co-activator-1α; TTC: 2,3,5-triphenyltetrazolium chloride.

Infarct volume was lower in 1,5-AF rats (68.0 ± 17.8 mm³) than in control rats (463 ± 15.9 mm³, *p* < 0.001; Figure 1A). Neurological score was better in 1,5-AF rats (0.5 ± 0.2) than in control rats (3.7 ± 0.2, *p* = 0.002; Figure 1B). Mortality after AIS was also lower in 1,5-AF rats (16.7%) than in control rats (44.4%, *p* = 0.036; Figure 1C). Thus, 1,5-AF reduced cerebral infarct volume in rats with AIS; it also reduced neurological deficits and mortality.

### Intraperitoneal injection of 1,5-AF activates the pAMPK/PGC-1α/BDNF pathway in rats with AIS

The protein level of pAMPK was higher in 1,5-AF rats than in control rats (*p* = 0.016; Figure 1D, 1E). Protein levels of PGC-1α and BDNF were also higher in 1,5-AF rats than in control rats (*p* = 0.010 and *p* = 0.002; Figure 1D, 1F, 1G). BDNF and PGC-1α immunoreactivity around lesions was higher in 1,5-AF rats than in control rats (*p* < 0.001 and *p* < 0.001; Figure 1H–1J). The expression of fibronectin type III domain-containing protein 5 (FNDC5), which is associated with the pAMPK/PGC-1α/BDNF pathway, was also investigated. Both FNDC5 protein levels and FNDC5 immunoreactivity around lesions were higher in 1,5-AF rats than in control rats (*p* = 0.043 and *p* = 0.003; Supplementary Figure 1A–1D).

### Intraperitoneal injection of 1,5-AF inhibits tumor necrosis factor-α (TNF-α) and microglial activation in rats with AIS

**Figure 2:**
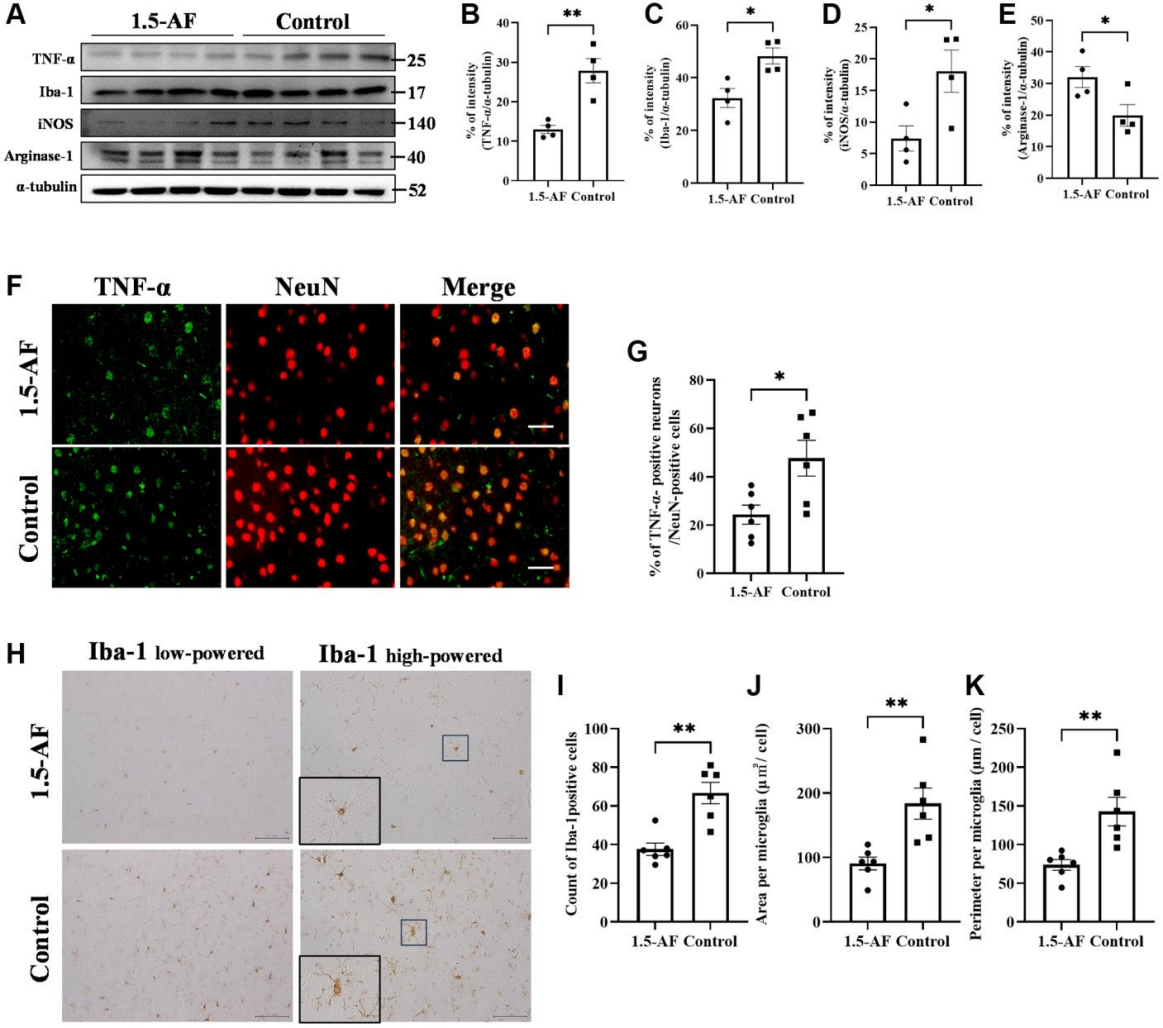
**1.5-AF inhibits inflammatory cytokines and microglial activation in an AIS model.** Immunoblotting results after AIS (1,5-AF: *n* = 4, control: *n* = 4) (**A**). Semiquantitative analysis of immunoblots revealed that protein levels of TNF-α (**B**), Iba1 (**C**), and iNOS (**D**) were lower in 1,5-AF rats than in control rats. The protein level of arginase-1 (**E**) was higher in 1,5-AF rats than in control rats. Fluorescent double-stained images of TNF-α and NeuN (neurons) in the penumbra region (1,5-AF: *n* = 6, control: *n* = 6) (**F**). The percentage of NeuN cells co-stained with TNF-α was lower in 1,5-AF rats than in control rats (**G**). Photomicrographs of Iba1 immunoreactivity (1,5-AF: *n* = 6, control: *n* = 6) (**H**). There were fewer activated microglia in 1,5-AF rats than in control rats (**I**). Both the area (**J**) and perimeter diameter (**K**) of activated microglia were smaller in 1,5-AF rats than in control rats. Data are shown as the mean ± standard error. ^*^p < 0.05, ^**^p < 0.01, ^***^p < 0.001. Scale bars: (**F**) 30 μm, (**H**) Iba1 low-powered: 100 μm, Iba1 high-powered: 50 μm. Abbreviations: 1,5-AF: 1,5-anhydro-D-fructose; AIS: acute ischemic stroke; Iba1: allograft inflammatory factor 1; iNOS: inducible nitric oxide synthase; TNF-α: tumor necrosis factor-α.

To evaluate brain inflammation, we examined the expression of TNF-α and the activation of allograft inflammatory factor 1 (Iba1). Protein levels of TNF-α and Iba1 were lower in 1,5-AF rats than in control rats (*p* = 0.004 and *p* = 0.0147; Figure 2A–2C). Protein levels of inducible nitric oxide synthase (iNOS; a marker of M1 microglia) were significantly lower in 1,5-AF rats than in control rats (*p* = 0.033; Figure 2A, 2D). In contrast, the protein level of arginase-1 (a marker of M2 microglia) was significantly higher in 1,5-AF rats than in control rats (*p* = 0.043; Figure 2A, 2E). Moreover, the percentages of TNF-α- and NeuN-positive cells around lesions were significantly higher in control rats than in 1,5-AF rats (*p* = 0.019; Figure 2F, 2G). Activated microglia were then evaluated by counting positive cell number, size, and perimeter. The number of activated microglia was significantly lower in 1,5-AF rats than in control rats (*p* = 0.001; Figure 2H, 2I). Microglial size and perimeter were also significantly smaller in 1,5-AF rats than in control rats (*p* = 0.005 and *p* = 0.006; Figure 2H, 2J, 2K).

### Oral ingestion of 1,5-AF prolongs survival in high-salt-water SHRSPs

**Figure 3:**
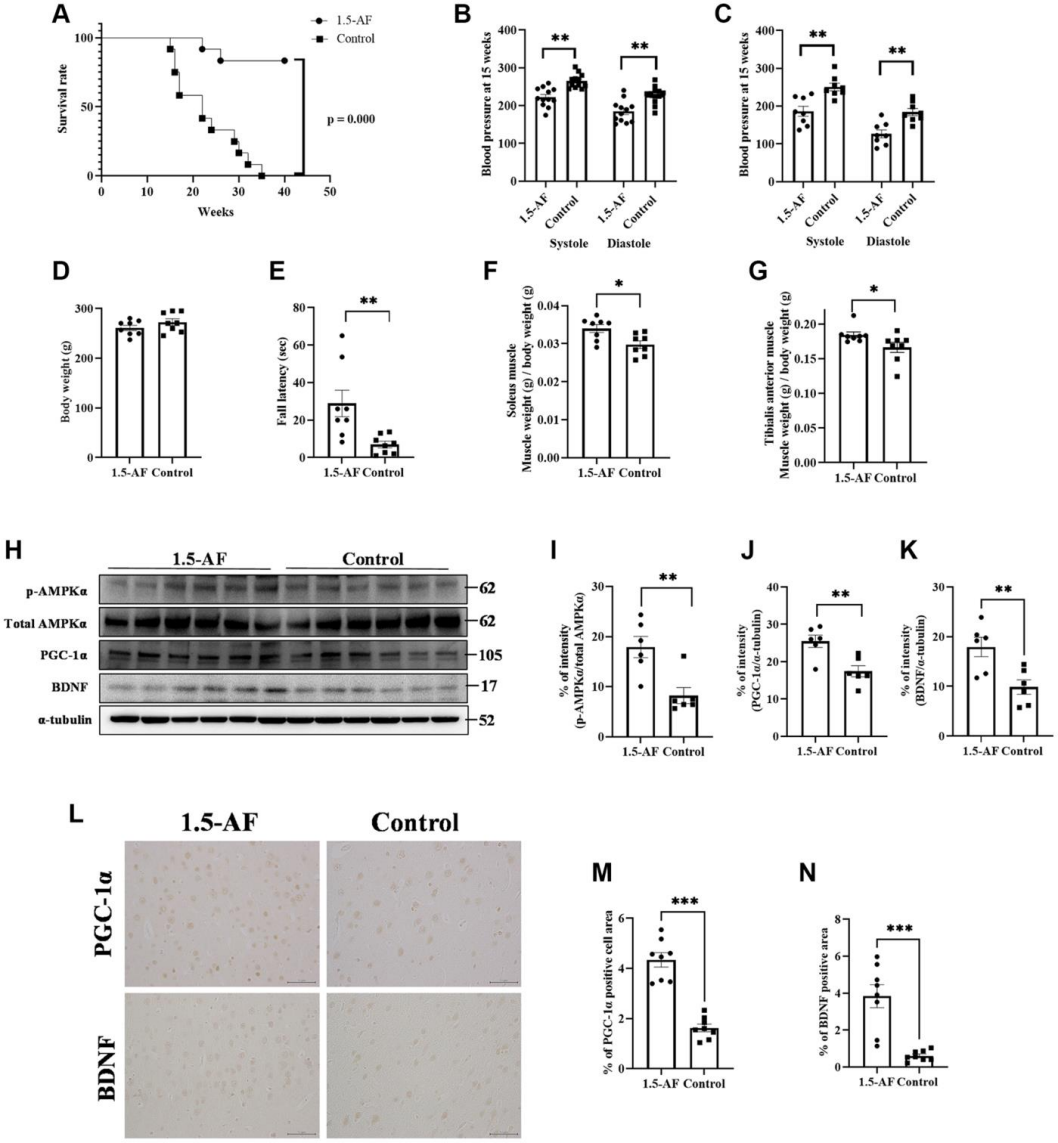
**1,5-AF activates pAMPK/PGC-1α/BDNF pathway and prolongs survival in high-salt-water SHRSPs.** Kaplan–Meier survival curves in 1,5-AF and control rats, according to log-rank test (**A**) (1,5-AF: *n* = 12, control: *n* = 12). 1,5-AF treatment reduced mortality. Blood pressure at 15 weeks in rats used for survival analysis (**B**) (1,5-AF: *n* = 12, control: *n* = 12) and in rats used for tissue collection (**C**) (1,5-AF: *n* = 8, control: *n* = 8). Blood pressure at 15 weeks of age was consistently lower in 1,5-AF rats than in control rats. Body weight (**D**) (1,5-AF: *n* = 12, control: *n* = 12), rotarod test results (**E**) (1,5-AF: *n* = 12, control: *n* = 12), soleus muscle weight (**F**) (1,5-AF: *n* = 12, control: *n* = 12), and tibialis anterior muscle weight (**G**) (1,5-AF: *n* = 12, control: *n* = 12). Body weight did not differ between groups. Rotarod tests revealed greater walking time in 1,5-AF rats. Soleus and tibialis anterior muscle weights were lower in control rats than in 1,5-AF rats. Immunoblot analysis (**H**) revealed that protein levels of pAMPK (**I**), PGC-1α (**J**), and BDNF (**K**) were higher in 1,5-AF rats than in control rats (1,5-AF: *n* = 6, control: *n* = 6). Photomicrographs of PGC-1α and BDNF immunoreactivity (1,5-AF: *n* = 8, control: *n* = 8) (**L**). PGC-1α (**M**) and BDNF (**N**) expression was greater in 1,5-AF rats than in control rats. Data are shown as the mean ± standard error. ^*^p < 0.05, ^**^p < 0.01, ^***^p < 0.001. Scale bar = 50 μm.

In the AIS model, 1,5-AF ameliorated cerebral infarction through an AMPK-mediated pathway. However, those rats were only assessed for 24 h post-ischemia; we presumed that a longer analysis would clarify whether continued 1,5-AF treatment could facilitate or accelerate recovery. Therefore, we evaluated SHRSPs that received consistent levels of saline intake throughout the experimental period. In the survival analysis, all rats in the control group reached the endpoint criteria by 35 weeks of age; in contrast, only two rats in the 1,5-AF group had died by that age (*p* < 0.001; Figure 3A). Notably, the first death in the control group occurred in a 15-week-old rat. At 15 weeks of age, systolic BP was higher in control rats (265.1 ± 5.5 mmHg) than in 1,5-AF rats (222.4 ± 7.2 mmHg, *p* < 0.001; Figure 3B); diastolic BP was also higher in control rats (227.6 ± 6.7 mmHg) than in 1,5-AF rats (184.7 ± 8.3 mmHg, *p* < 0.001; Figure 3B). Although BP measurements were performed before the rats reached 15 weeks of age, they did not significantly differ between groups (data not shown). Because the first rat died at 15 weeks of age, tissue collection was performed in rats at that age. Among the rats used for tissue collection (distinct from the rats used for survival analysis; please see Methods for details), systolic BP (1,5-AF: 186.3 ± 13.1 mmHg, control: 250.1 ± 9.8 mmHg, *p* = 0.001; Figure 3C) and diastolic BP (1,5-AF: 126.6 ± 10.5 mmHg, control: 184.3 ± 9.3 mmHg, *p* = 0.002; Figure 3C) were lower in 1,5-AF rats than in control rats.

Body weights were similar in 1,5-AF rats (260.9 ± 5.3 g) and control rats (272.5 ± 6.9 g, *p* = 0.21; Figure 3D). Walking time in the rotarod test was greater in 1,5-AF rats (28.8 ± 7.1 s) than in control rats (7.0 ± 2.5 s, *p* = 0.009; Figure 3E). At 15 weeks of age, soleus muscle weight was lower in control rats (0.03% ± 0.001%) than in 1,5-AF rats (0.034% ± 0.001%, *p* = 0.011; Figure 3F). Tibialis anterior muscle weight was also lower in control rats (0.172% ± 0.0078%) than in 1,5-AF rats (0.184% ± 0.0042%, *p* = 0.037; Figure 3G).

### Oral ingestion of 1,5-AF activates the pAMPK/PGC-1α/BDNF pathway in high-salt-water SHRSPs

The protein level of pAMPK was higher in 1,5-AF rats than in control rats (*p* = 0.009; Figure 3H, 3I). Protein levels of PGC-1α and BDNF were also higher in 1,5-AF rats than in control rats (*p* = 0.005 and *p* = 0.008; Figure 3H–3K). Similarly, PGC-1α and BDNF immunoreactivity in the left and right cortical regions was higher in 1,5-AF rats than in control rats (*p* < 0.001 and *p* < 0.001; Figure 3L–3N). The expression of FNDC5 (associated with the pAMPK/PGC-1α/BDNF pathway) was also evaluated. Both the protein levels of FNDC5 and the percentages of FNDC5 immunoreactivity in left and right cortical regions were higher in 1,5-AF rats than in control rats (*p* < 0.001 and *p* < 0.001; Supplementary Figure 1E–1H).

Importantly, SHRSP samples were not collected or analyzed after survival measurement because all SHRSPs in the control group were in poor condition for tissue collection at the time of death (≤35 weeks of age); thus, we could not compare tissues between the 1,5-AF and control groups.

### Oral ingestion of 1,5-AF inhibits TNF-α and microglial activation in high-salt-water SHRSPs

**Figure 4:**
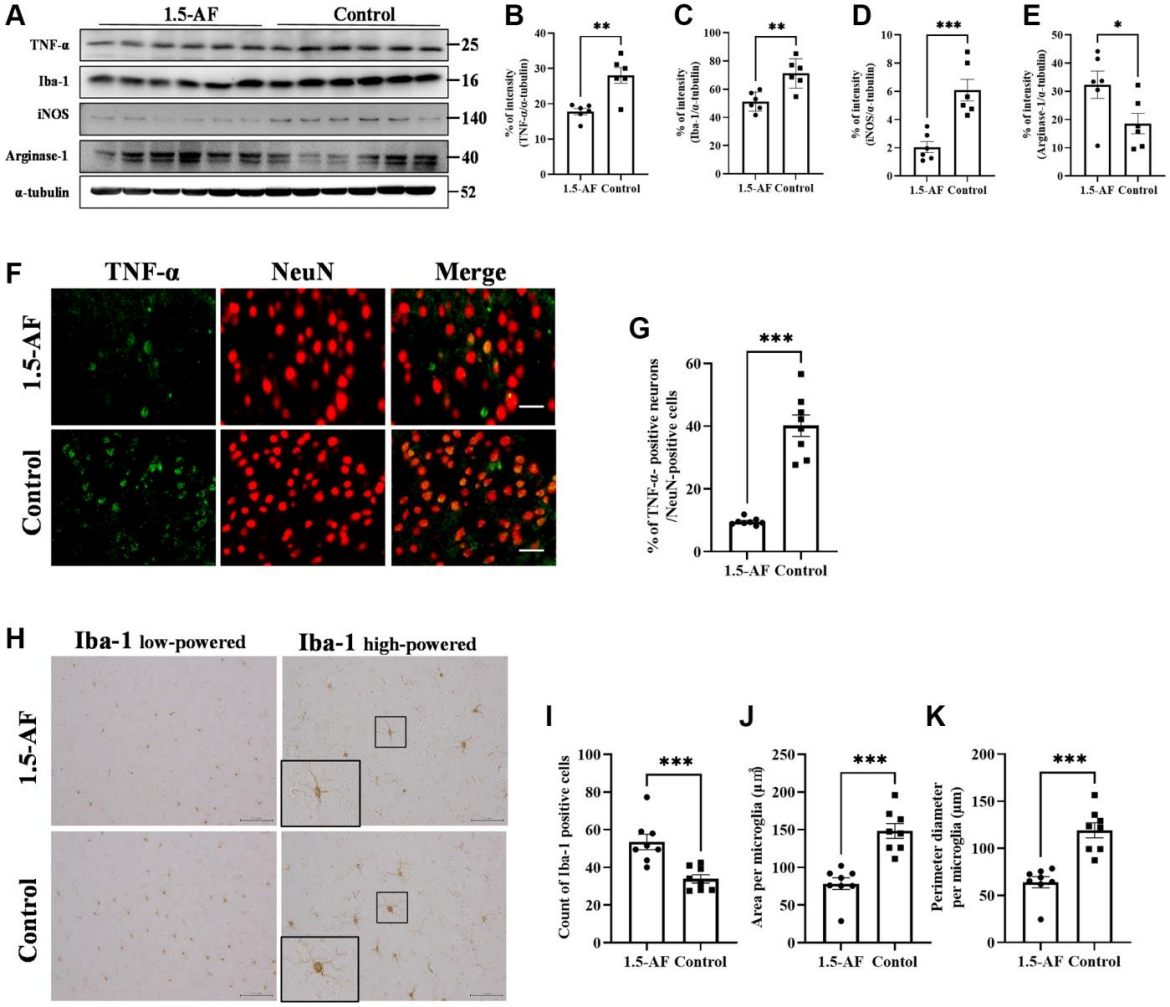
**1.5-AF inhibits inflammatory cytokines and microglial activation in high-salt-water SHRSPs.** Immunoblot analysis (**A**) revealed that protein levels of TNF-α (**B**), Iba1 (**C**), and iNOS (**D**) were lower in 1,5-AF rats than in control rats. The protein level of arginase-1 (**E**) was higher in 1,5-AF rats than in control rats (1,5-AF: *n* = 6, control: *n* = 6). Fluorescent double-stained images of TNF-α and NeuN (neurons) in the motor cortex (1,5-AF: *n* = 8, control: *n* = 8) (**F**). The percentage of NeuN cells that were co-stained with TNF-α was lower in 1,5-AF rats than in control rats (**G**). Photomicrographs of Iba1 immunoreactivity (1,5-AF: *n* = 8, control: *n* = 8) (**H**). There were fewer activated microglia in 1,5-AF rats than in control rats (**I**). Both the area (**J**) and perimeter (**K**) of activated microglia were smaller in 1,5-AF rats than in control rats. Data are shown as the mean ± standard error. ^*^p < 0.05, ^**^p < 0.01, ^***^p < 0.001. Scale bar: (**F**) 30 μm, (**H**) Iba1 low-powered: 100 μm, Iba1 high-powered: 50 μm. Abbreviations: 1,5-AF: 1,5-anhydro-D-fructose; Iba1: allograft inflammatory factor 1; iNOS: inducible nitric oxide synthase; SHRSPs: stroke-prone spontaneously hypertensive rats; TNF-α: tumor necrosis factor-α.

To evaluate brain inflammation, we examined TNF-α expression and Iba1 activation. Protein levels of TNF-α and Iba1 were lower in 1,5-AF rats than in control rats (*p* = 0.002 and *p* = 0.003; Figure 4A–4C). The protein level of iNOS, a marker of M1 microglia, was also significantly lower in 1,5-AF rats than in control rats (*p* = 0.001; Figure 4A, 4D). However, the protein level of arginase-1, a marker of M2 microglia, was significantly higher in 1,5-AF rats than in control rats (*p* = 0.046; Figure 4A, 4E). The percentages of TNF-α- and NeuN-positive cells were then evaluated. The percentages of TNF-α- and NeuN-positive cells around the cortex were significantly lower in 1,5-AF rats than in control rats (*p* < 0.001; Figure 4F, 4G). Activated microglia were evaluated by counting positive cells (i.e., microglia that had projections), measuring cell body area, and measuring the circumference of individual cell bodies. The number of activated microglia was significantly lower in 1,5-AF rats than in control rats (*p* < 0.001; Figure 4H, 4I). Furthermore, both the area and circumference of microglial cell bodies were significantly lower in 1,5-AF rats than in control rats (*p* < 0.001 and *p* < 0.001; Figure 4H, 4J, 4K).

### Oral ingestion of 1,5-AF may maintain age-related motor cognitive function in SAMP8 mice

**Figure 5:**
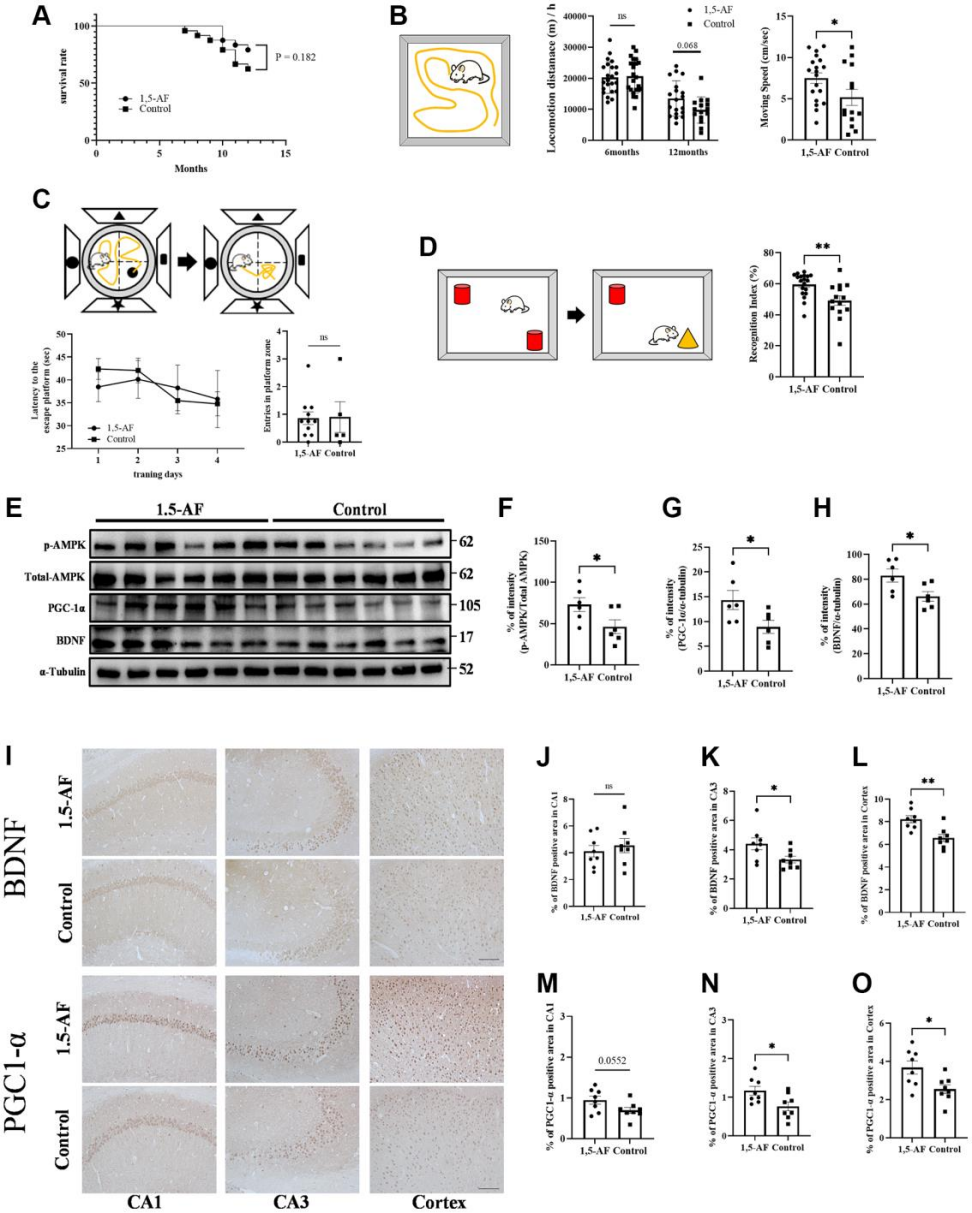
**1,5-AF activates pAMPK/PGC-1α/BDNF pathway in SAMP8 mice to maintain activity and cognitive function.** Kaplan–Meier survival curves in 1,5-AF and control mice, according to log-rank test (**A**); survival did not significantly differ between groups (*p* = 0.18) (1,5-AF: *n* = 19, control: *n* = 14). Open field test revealed greater locomotor activity and movement speed per unit time in 1,5-AF mice than in control mice (**B**) (1,5-AF: *n* = 19, control: *n* = 15). Morris water maze analysis showed no significant differences between groups in time to reach the platform or number of platform crossings (**C**) (1,5-AF: *n* = 11, control: *n* = 6). Novel object recognition test revealed higher orientation to a novel object in 1,5-AF mice (**D**) (1,5-AF: *n* = 19, control: *n* = 15). Immunoblot analysis (**E**) revealed that protein levels of pAMPK (**F**), PGC-1α (**G**), and BDNF (**H**) were higher in 1,5-AF mice than in control mice (1,5-AF: *n* = 6, control: *n* = 6). Representative image of BDNF and PGC1-α staining in the cortex and hippocampal CA1 and CA3 regions (**I**). BDNF immunoreactivity in the CA3 (**J**) and cortex (**K**) was higher in 1,5-AF mice than in control mice, whereas immunoreactivity in the CA1 (**L**) did not differ (1,5-AF: *n* = 6, control: *n* = 6). PGC1-α immunoreactivity in the CA3 (**M**) and cortex (**N**) was also higher in 1,5-AF mice than in control mice, whereas immunoreactivity in the CA1 (**O**) did not differ (1,5-AF: *n* = 6, control: *n* = 6). Data are shown as the mean ± standard error. ^*^p < 0.05, ^**^p < 0.01. Scale bar = 100 μm (**I**). Abbreviations: 1,5-AF: 1,5-anhydro-D-fructose; BDNF: brain-derived neurotrophic factor; pAMPK: phosphorylated 5′-adenosine monophosphate-activated protein kinase; PGC-1α: peroxisome proliferator-activated receptor-γ co-activator-1α; SAMP8: senescence-accelerated mouse-prone 8.

Our findings in acute and chronic models of experimental stroke revealed that 1,5-AF causes beneficial effects via the AMPK/PGC-1α/BDNF pathway. We sought to validate these findings in SAMP8 mice, thus clarifying the effects of 1,5-AF on aging-related declines in physical and cognitive functions. The mean lifespan of SAMP8 mice is 9.7 months [26]. Here, SAMP8 mice were bred until 12 months of age to allow sufficient aging; debilitated mice with weight loss began to appear at 7 months of age in the control group and at 10 months of age in the 1,5-AF group. Consistent intake of 1,5-AF was confirmed in all 1,5-AF mice. At 12 months of age, eight of 22 mice in the control group and four of 23 mice in the 1,5-AF group were euthanized in accordance with the humane endpoint protocol (*p* = 0.18; Figure 5A).

To determine aging-related decline in locomotor activity, we assessed locomotor activity by open field (OF) tests when mice were 6 and 12 months of age. Because we had previously found no decline in locomotor activity among 5-month-old mice [9], we opted to perform the first measurement at 6 months of age. Here, both groups showed lower motor activity at 12 months of age than at 6 months of age (1,5-AF: 6 months: 202.63 ± 10.65 m/h, 12 months: 134.58 ± 13.09 m/h; control: 6 months: 208.67 ± 11.81 m/h, 12 months: 95.51 ± 11.56 m/h; Figure 5B). 1,5-AF intake mitigated aging-related decline in exercise activity (group effect: *p* = 0.21, time effect: *p* < 0.001, interaction effect: *p* = 0.037). At 12 months of age, 1,5-AF mice tended to have higher locomotor activity than control mice (Sidak's multiple comparisons: *p* = 0.068). Mean movement velocity was greater in 1,5-AF mice than in control mice (1,5-AF: 7.51 ± 0.63 cm/s, control: 5.18 ± 0.97 cm/s, *p* = 0.045; Figure 5B).

Morris water maze (MWM) analysis was conducted to evaluate cognitive function in 12-month-old mice. Among control mice, the escape latencies were 38.49 ± 3.29 s on day 1 and 35.82 ± 6.25 s on day 4; among 1,5-AF mice, these values were 42.41 ± 2.27 s and 34.79 ± 2.64 s, respectively. There were no significant differences between groups (group effect: *p* = 0.62, time effect: *p* = 0.12, interaction effect: *p* = 0.89; Figure 5C). For 1,5-AF and control mice, the numbers of platform crossings were 0.86 ± 0.22 and 0.9 ± 0.55, respectively; they did not significantly differ between groups (*p* = 0.64; Figure 5C).

Novel object recognition (NOR) tests were conducted in 12-month-old mice to evaluate recognition memory. Discrimination index values were greater among 1,5-AF mice than among control mice (1,5-AF: 59.50% ± 1.75%, control: 49.13% ± 3.11%, *p* < 0.001; Figure 5D).

### Oral ingestion of 1,5-AF activates the pAMPK/PGC-1α/BDNF pathway in SAMP8 mice

Among 12-month-old SAMP8 mice, the protein level of pAMPK was higher in 1,5-AF mice than in control mice (*p* = 0.046; Figure 5E, 5F). Protein levels of PGC-1α and BDNF were also higher in 1,5-AF mice than in control mice (PGC-1α: *p* = 0.008, BDNF: *p* = 0.006; Figure 5E, 5G, 5H). Moreover, we quantified BDNF and PGC1-α immunoreactivity in the hippocampus (CA1 and CA3 regions) and cortex (Figure 5I). BDNF immunoreactivity in the CA1 did not significantly differ between 1,5-AF and control mice (*p* = 0.54; Figure 5I, 5J). However, in the CA3 and cortex, BDNF immunoreactivity was higher in 1,5-AF mice than in control mice (CA3: *p* = 0.037, cortex: *p* = 0.002; Figure 5I, 5K, 5L). PGC1-α immunoreactivity in the CA1 tended to be higher in 1,5-AF mice than in control mice (*p* = 0.055; Figure 5I, 5M). Moreover, PGC1-α immunoreactivity in the CA3 and cortex was higher in 1,5-AF mice than in control mice (CA3: *p* = 0.025, cortex: *p* = 0.016; Figure 5I, 5N, 5O). FNDC5 immunoreactivity in the CA1, CA3, and cortex was higher in 1,5-AF mice than in control mice (CA1: *p* = 0.004, CA3: *p* < 0.001, cortex: *p* = 0.004; Supplementary Figure 1H–1L).

### Effects of oral 1,5-AF ingestion on neuroinflammation in SAMP8 mice

**Figure 6:**
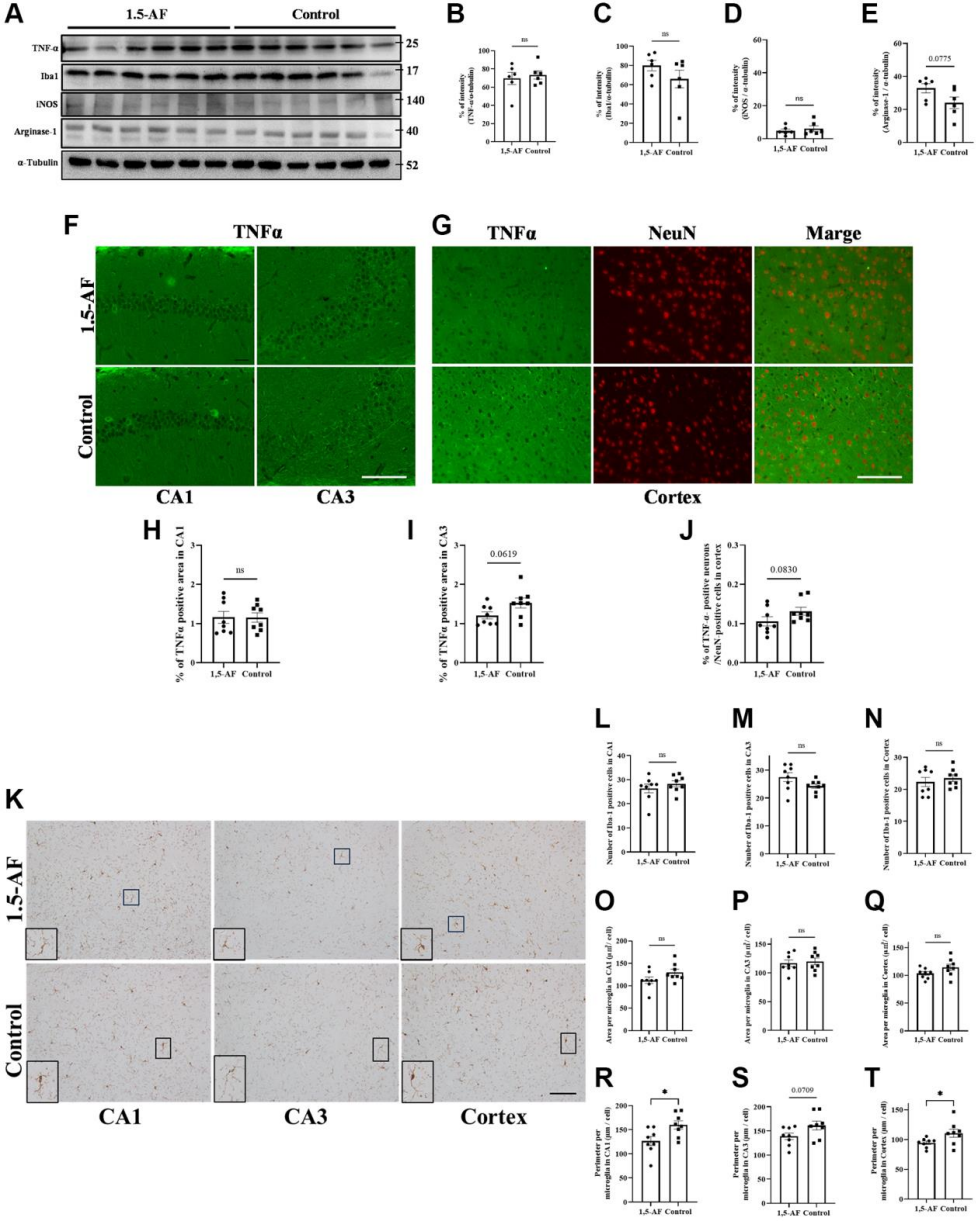
**Effect of 1.5-AF on brain inflammation in SAMP8 mice.** Immunoblot analysis (**A**) revealed that protein levels of TNF-α (**B**), Iba1 (**C**), iNOS (**D**), and arginase-1 (**E**) were not significantly different between 1,5-AF and control mice (1,5-AF: *n* = 6, control: *n* = 6). TNF-α staining in the hippocampal CA1 and CA3 regions (**F**). Co-stained images of NeuN (neurons; red) and TNF-α (green) in the Cortex (**G**). In all regions, TNF-α expression did not differ significantly between the two groups (**H**–**J**) (1,5-AF: *n* = 6, control: *n* = 6). Similarly, when analyzing the Iba1 staining of the cortex and hippocampal CA1 and CA3 regions (**K**); the number (**L**–**N**), and cell size (**O**–**Q**) of Iba1-positive microglia did not significantly differ between 1,5-AF and control mice. In contrast, microglial perimeters (PE) were significantly smaller in CA1 (**R**), unchanged in CA3 (**S**), and smaller in Cortex (**T**) at 1,5-AF mice compared to Control mice. (1,5-AF: *n* = 6, control: *n* = 6). Data are shown as the mean ± standard error. ^*^p < 0.05. Scale bar = 100 μm (**F**, **G**, **K**). Abbreviations: 1,5-AF: 1,5-anhydro-D-fructose; Iba1: allograft inflammatory factor 1; iNOS: inducible nitric oxide synthase; SAMP8: senescence-accelerated mouse-prone 8; TNF-α: tumor necrosis factor-α.

Protein levels of TNF-α, Iba1, and iNOS were not significantly different between 1,5-AF and control mice (TNF-α: *p* = 0.64, Iba1: *p* = 0.21, iNOS: *p* = 0.49; Figure 6A–6D). However, arginase-1 protein levels tended to be higher in 1,5-AF mice than in control mice (*p* = 0.075; Figure 6A, 6E). Next, we attempted to assess the percentages of TNFα- and NeuN-positive cells using fluorescent staining. However, the hippocampus had a dense neuronal population, which made it difficult to distinguish between TNFα- and NeuN-positive cells. We therefore counted both TNFα- and NeuN-positive cells in the cortex but evaluated the immunoreactivity of TNFα alone in the CA1 and CA3. In 1,5-AF mice, there was a trend toward lower TNFα expression in the CA3 and cortex compared with control mice (CA1: *p* = 0.98, CA3: *p* = 0.062, cortex: *p* = 0.083; Figure 6F–6J). In addition, the number, size, and perimeter of activated microglia in the CA1, CA3, and cortex were evaluated. Microglial number and size were not significantly different between 1,5-AF and control mice (Figure 6K, 6L–6Q). However, microglial perimeters in the CA1 and cortex were lower in 1,5-AF mice than in control mice (CA1: *p* = 0.019, CA3: *p* = 0.070, cortex: *p* = 0.046; Figure 6R–6T).

## DISCUSSION

**Figure 7:**
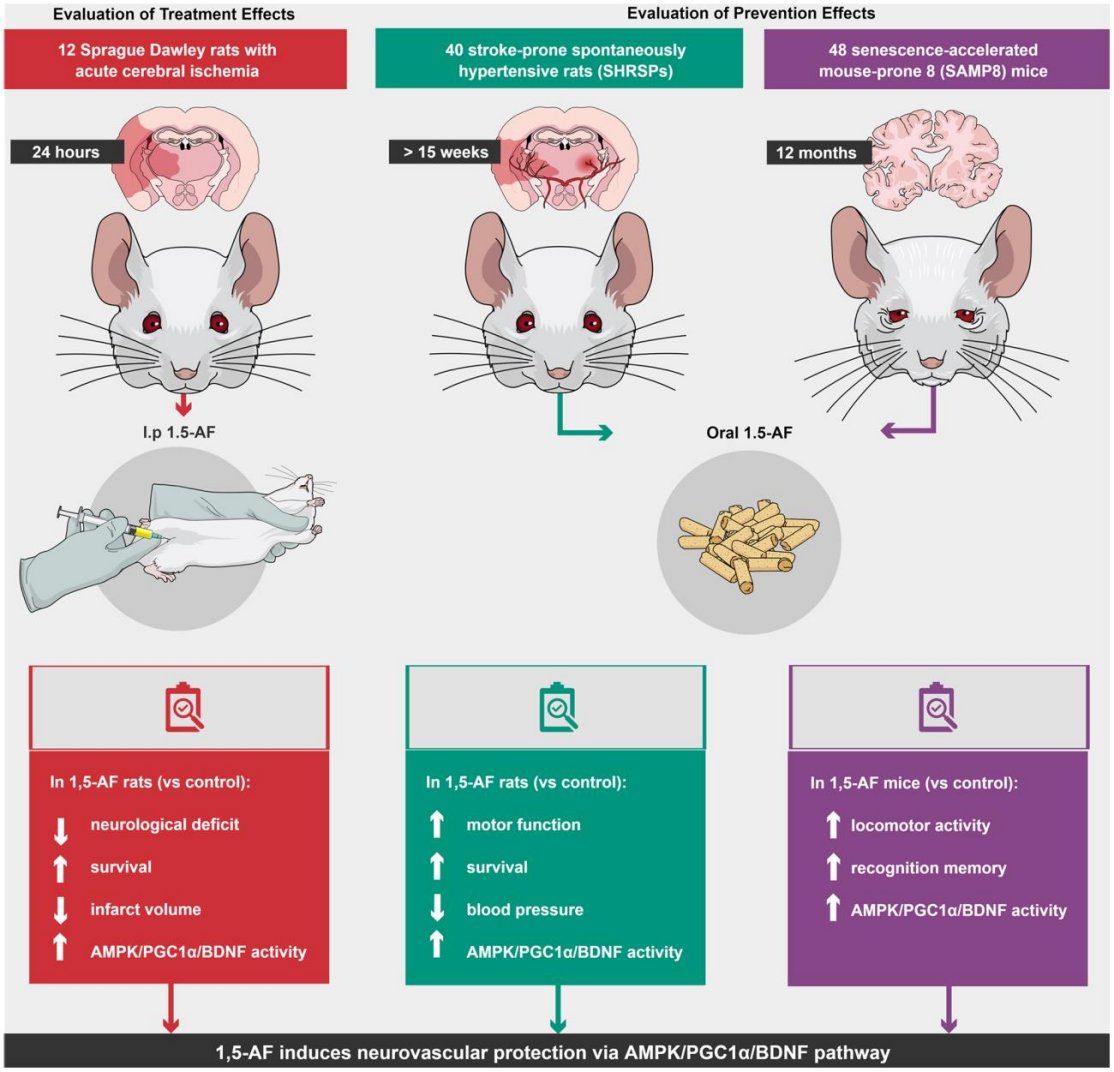
**Schema of various effects and identical mechanisms of 1,5-AF in models of aging-associated brain diseases.** Abbreviations: 1,5-AF: 1,5-anhydro-D-fructose; AMPK: 5′-adenosine monophosphate-activated protein kinase; BDNF: brain-derived neurotrophic factor; PGC-1: peroxisome proliferator-activated receptor-γ co-activator-1.

Here, we examined the behavioral and molecular effects of 1,5-AF in multiple animal models of human aging-associated brain diseases. We found that 1,5-AF activates AMPK and may induce brain damage tolerance via the downstream PGC-1α/BDNF pathway (Figure 7). The main mechanisms underlying the 1,5-AF-induced improvements in our animal models may comprise neurovascular protection and plasticity [27] and ischemic tolerance [28] caused by BDNF via AMPK/PGC-1α activity. In this study, we had several important findings.

First, concerning the AIS model, previous reports have shown that BDNF induces a dose- and time-dependent increase in tissue plasminogen activator/plasminogen expression [29]. The reduction in cerebral infarct volume by 1,5-AF, which led to improved neurological function in our rats, may be related to enhancement of the fibrinolytic system by BDNF. This effect may occur along with the aforementioned neurovascular protection, neurovascular plasticity, and ischemia tolerance.

Second, in SHRSPs, 1,5-AF treatment reduced the incidence of stroke because of the BP-lowering effect of 1,5-AF; muscle mass was also preserved in these rats. Both BP reduction and muscle mass preservation are expected to prolong survival. However, BDNF can increase BP [30]; the hypotensive effect of 1,5-AF in SHRSPs may thus be induced by another AMPK signaling pathway [4]. We also observed a change in muscle weight among SHRSPs. Typically, SHRSPs exhibit weight loss and a decline in physical function during BP elevation and stroke onset [31]. In our SHRSPs, weight loss was greater in 1,5-AF rats than in control rats; however, 1,5-AF rats maintained skeletal muscle weight. Weight loss in 1,5-AF rats was presumably caused by fat loss rather than the poor physical function associated with increased BP. Our findings are consistent with previous results. Enhanced BDNF production in the brain is induced by prolonged BDNF infusion [28] or spreading depolarization [32]. Moreover, a clinical trial showed that lower BDNF levels are associated with increased risk of stroke/transient ischemic attack [33].

Third, in SAMP8 mice, 1,5-AF treatment led to increased spontaneous locomotor activity and reduced memory impairment. These effects may involve the reversal of cognitive deficits through increased PGC-1α and BDNF expression in the brain, particularly in the hippocampus (CA3). Our OF test findings in SAMP8 mice have behavioral implications. These mice become less active with age [9]. Our OF test results suggest that 1,5-AF intake prevented SAMP8 mice from experiencing aging-related decline in locomotor activity. Our findings are consistent with previous reports. PGC-1α expression is lower in SAMP8 mice than in corresponding control mice in both the cortex and hippocampus; furthermore, PGC-1α activation-mediated control and maintenance of mitochondrial function have therapeutic potential for aging-related pathologies [21]. We recently reported that 1,5-AF activates PGC-1α via AMPK, with potential mitochondrial biogenesis and cytoprotective effects in rotenone-treated PC12 cells [12]. The maintenance of mitochondrial function may help to prevent or delay aging-related diseases; PGC-1α also has diverse effects on other cellular processes, which may help to prevent age-related pathologies. Additionally, serum BDNF concentrations decline with age [34]. Increased BDNF levels contribute to the survival of neuronal [35] and endothelial [36] cells, synaptic consolidation [37], and cognitive improvement in AD [38]. Higher BDNF levels are also associated with fewer white matter lesions and better visual memory performance [33]. In our study, BDNF levels were increased in CA3 and the cortex after 1,5-AF treatment in SAMP8 mice; they did not differ in CA1. Both CA1 and CA3 regions are affected by aging [39]. Although anti-aging interventions may be less effective in CA1 than in CA3, there is no clear explanation for such findings [40]; further investigations are needed. Notably, 1,5-AF treatment did not alter MWM behavior in SAMP8 mice, although it altered NOR test results. This is presumably because SAMP8 mice had difficulty swimming freely in water. Finally, in both SHRSPs and SAMP8 mice, differences in immunoblot results could have arisen from differences in 1,5-AF ingestion; however, all animals received the same amount of 1,5-AF.

It is unclear whether 1,5-AF-related improvement in multiple animal models of aging-associated brain diseases is entirely related to AMPK activation; AMPK regulation *in vivo* remains poorly understood. Konagaya et al. [41] developed transgenic mice expressing a highly sensitive fluorescence resonance energy transfer-based biosensor for AMPK; these mice may help to inspect the effects of 1,5-AF on AMPK activation.

Consideration of other AMPK activators can clarify the benefits of 1,5-AF. Metformin and the AMPK agonist 5-aminoimidazole-4-carboxamide ribonucleotide (AICAR) are AMPK activators. Moreover, 1,5-AF, metformin [42], and AICAR [43] enhance BDNF expression. Metformin is used clinically; it can reduce stroke incidence in high-risk populations [44]. However, AMPK-targeted preventive therapy has some limitations. First, it is unclear whether AMPK plays a neuroprotective role; it may have negative effects [45]. Second, few AMPK activators have been used in clinical treatment, and few clinical trials have focused on AMPK [7]. Metformin and AICAR also have some adverse effects. The most common side effects of metformin are gastrointestinal disturbances, metallic taste, and vitamin B12 malabsorption [46]. Furthermore, the effects of metformin differ on the basis of administration method: in mice with AIS, intracerebroventricular administration led to excessive AMPK activation, increased infarct foci, and worsened neurological deficits compared with intraperitoneal administration [47]. AICAR has a short half-life after intravenous administration and poor bioavailability after oral ingestion; because it increases blood levels of uric acid and lactic acid, it is unsuitable for long-term use [48]. Moreover, excessive AMPK activation could disrupt cell proliferation; in the hypothalamus, excessive AMPK activation can increase food intake, which is unsuitable for individuals with obesity or type 2 diabetes [49]. Thus, metformin and AICAR are not clinically ideal for preventing aging-associated brain diseases.

In western blot and immunohistochemical assays, FNDC5 (which is involved in the AMPK/PGC-1α/BDNF pathway) had increased expression after 1,5-AF intake in both the AIS model and SHRSPs. Similarly, although we were unable to conduct western blot assays in SAMP8 mice because of a lack of samples, immunohistochemical staining in these mice also revealed the increased expression of FNDC5. A trend toward increased FNDC5 expression was observed in all three models, indicating that 1,5-AF may increase FNDC5 expression. Thus, although 1,5-AF induction of FNDC5 expression needs to be further investigated, our FNDC5 results were consistent with AMPK/PGC-1α/BDNF pathway activation.

Finally, exercise may have beneficial effects on aging-associated brain diseases [1] by activating the AMPK/PGC-1α/BDNF pathway [43], indicating that some compounds can mimic the central effects of exercise. 1,5-AF, which acts on the AMPK/PGC-1α/BDNF pathway, may offer a valuable option for many individuals, regardless of exercise ability.

The present study had some limitations; we had a small number of rats with AIS, a model that has very high mortality. Thus, SPSS Sample Power software was used to minimize the number of rats euthanized. Furthermore, additional evaluation of compound C—a small molecule compound commonly used as an AMPK inhibitor—might have clarified whether the effects of 1,5-AF were related to AMPK activation. However, we sought to limit the number of animals euthanized. Moreover, SHRSPs and SAMP8 mice have systemic alterations that limit comparisons of their tissue with otherwise healthy brain tissue in aging humans. Thus, we did not perform compound C experiments in SHRSPs or SAMP8 mice.

In conclusion, despite increasing comprehension of aging-associated brain diseases, further research is needed concerning cognitive impairment among the increasing population of older adults worldwide [1]. Our results suggest that 1,5-AF has preventive effects on aging-associated brain diseases via the AMPK/PGC-1α/BDNF pathway; these findings may encourage further clinical trials of 1,5-AF.

## MATERIALS AND METHODS

### Experimental design: AIS model

The experimental animal protocol was approved by the Institutional Animal Care and Use Committee at Kagoshima University, Kagoshima, Japan (Ethics approval number: MD18078, Approval date: 21 Nov 2018).

### Animals and stroke model induction: AIS model

**Figure 8:**
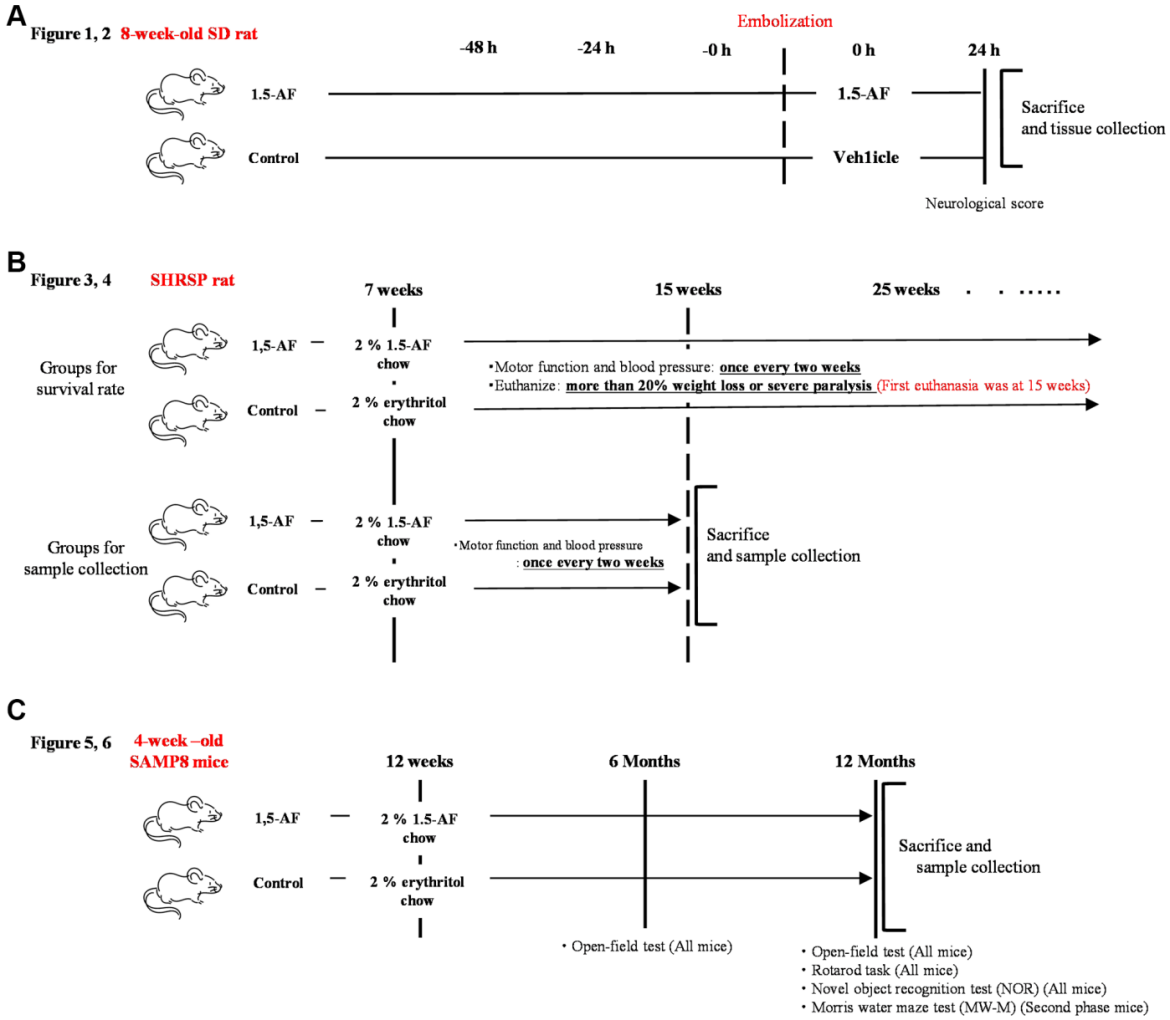
**Overview of experimental protocols.** (**A**) Sprague–Dawley rats were used to show effects of intraperitoneal 1,5-AF on thrombotic stroke. (**B**) SHRSPs were used to show effects of oral 1,5-AF on hypertension-related spontaneous stroke. (**C**) SAMP8 mice were used to show effects of oral 1,5-AF on aging-associated brain diseases. Abbreviations: 1,5-AF: 1,5-anhydro-D-fructose; SAMP8: senescence-accelerated mouse-prone 8; SHRSPs: stroke-prone spontaneously hypertensive rats.

The experimental protocol is depicted in Figure 8A. Thromboembolic ischemia was induced in the middle and posterior cerebral arteries via homologous blood clots in 41 Sprague–Dawley rats (8 weeks old, 290–310 g, male) (KBT Oriental, Itabashi, Tokyo, Japan), as previously described [50, 51].

Briefly, anesthesia was induced via isoflurane inhalation. A 24-gauge catheter (SURFLO Flash^®^; Terumo, Tokyo, Japan) was introduced into the left internal carotid artery; a 5-mm thrombus (volume, 3.6 mm^3^) was then forced through the catheter into the artery. Next, the catheter containing the thrombus was connected to a syringe containing saline solution. The clot and saline were securely inserted into the distal internal carotid, anterior cerebral (proximal portion), middle cerebral, and posterior cerebral arteries. Each rat then had a neurological score of four (indicating spontaneous right circling) according to a previously described five-point motor function scale [52]. Deaths were recorded; euthanasia endpoints included the appearance of imminent death and the onset of severe epileptic seizures that could not be evaluated by neurological examination. Each rat was assessed for neurological impairment after awakening and 24 h after cerebral ischemia induction.

As in our previous studies [50, 51], we did not perform cerebral blood flow monitoring. Shimamura et al. [53] reported that cerebral blood flow monitoring is not required for acute ischemic stroke models; moreover, dissection of the temporal muscle can cause masticatory dysfunction and poor nutrition. Additionally, the solid clot was visible in the target arteries in the present study.

We used rats with neurological scores of three or four after awakening, in accordance with a previously described five-point motor function scale [52].

### 1,5-AF administration: AIS model

After cerebral ischemia induction, each rat was randomly assigned to one of two groups. Rats in the control group received an intra-abdominal injection of saline (0.9% NaCl) immediately after thromboembolism. Rats in the 1,5-AF group received an intra-abdominal injection of saline plus 1,5-AF (SUNUS, Kagoshima, Japan) immediately after thromboembolism; this was followed by an intra-abdominal injection of saline.

### Sample size calculation and tissue collection: AIS model

Surviving rats were studied 24 h after cerebral ischemia induction. In accordance with the IMPROVE (Ischemia Models: Procedural Refinements of *In vivo* Experiments) 30 guidelines. A randomization protocol was used to ensure that each cage contained two groups of rats. To eliminate cage effects, rats from different groups were placed in the same cage. Before the study began, we performed power calculations using SPSS Sample Power (IBM, Armonk, NY, USA) to determine the appropriate sample size. In accordance with our previous reports [50, 51], we assumed that the effect size for infarct volume would be 0.8–1.0. To detect differences with 80% power and one-tailed α = 0.05 using analysis of variance, each group required 4–5 rats. To adjust for deaths during treatment, we included six rats per group. We designed our model so that each group contained ≥6 surviving rats at 24 h after cerebral ischemia induction; thus, the number of rats differed among groups. There were 12 and six rats in the control and 1,5-AF groups, respectively. The mortality rate of the stroke model used in this study was 50%, whereas the 1,5-AF group had a 0% mortality rate; thus, there were six rats per group.

Rats were euthanized, and their brains were excised at 24 h after cerebral ischemia induction, as previously described [50, 51]. Each brain, including the region of ischemia, was subjected to histological, immunohistochemical, and 2,3,5-triphenyltetrazolium chloride (TTC) analyses.

### Experimental design: SHRSPs

The experimental animal protocol was approved by the Institutional Animal Care and Use Committee at Kagoshima University, Kagoshima, Japan (Ethics approval number: MD18055, Approval date: 10 Oct 2018).

### Animals: SHRSPs

The experimental protocol is depicted in Figure 8B. In total, 40 SHRSP/Izm rats (7 weeks old, 200–240 g, male) (SLC Animal Supply, Hamamatsu, Shizuoka, Japan) were used in this study. The rats were housed in pairs in a temperature-controlled environment at 22.0 ± 1.0°C on a 12-h light/dark cycle; food and saline were freely available. In SHRSPs, a high-salt diet induces a rapid increase in BP and accelerates stroke onset [54]. Thus, SHRSPs ≥7 weeks of age were provided with 0.9% saline solution (Otsuka Pharmaceutical, Osaka, Japan), instead of water, to induce early stroke and shorten the experiment. Each group contained 40 rats, in accordance with the work of Watanabe et al. [55].

### 1,5-AF administration: SHRSPs

SHRSPs were fed a plant polysaccharide-based diet (Oriental Yeast, Itabashi, Tokyo, Japan) with water (0.9% saline) plus 2% 1,5-AF (SUNUS) or 2% erythritol (reference supplement), as described by Ito et al. [11]; rats were permitted free access to food and saline.

SHRSPs were randomly divided into two groups (*n* = 20 each): 1,5-AF and control (erythritol). In each group, 12 rats were used for survival assessment, and eight rats were used for tissue collection. Body weight, motor function, and BP were measured at 2-week intervals (intakes were not measured, but all rats received 5 g food daily); rats were assessed until 15 weeks of age, when they began to die of convulsions. Samples were collected at 15 weeks of age because death was increasingly common after that point; it was thus difficult to ensure equal numbers of rats among groups.

### Motor function test

Motor function was assessed as described by Otsuka et al. [56], using a rotarod test (MK-670, Muromachi Kikai, Chuo, Tokyo, Japan). Rotation speed was increased every 6 s in 2.5-rpm increments, from 0 rpm to 25 rpm. Each rat completed three trials; the longest latency to fall was used for analysis.

### BP measurement and survival analysis

BP measurements were conducted once weekly, beginning when rats were 7 weeks old, using the tail-cuff microsensor device (model MK-2000A; Muromachi Kikai) employed by Bland et al. [57]. BP was measured as described by Kato et al. [58].

Survival was assessed as follows. Rats were observed twice daily; when they exhibited significant weight loss (>20%) or severe paralysis, euthanasia was performed as previously described [50, 51].

### Experimental design: SAMP8 mice

The experimental animal protocol was approved by the Institutional Animal Care and Use Committee at Kagoshima University, Kagoshima, Japan (Ethics approval number: MD18026, Approval date: 10 Jul 2018).

### Animals: SAMP8 mice

In total, 48 SAMP8 mice (4 weeks old, male) were obtained from SLC Animal Supply in two phases and used for experiments beginning at 12 weeks of age (body weight: 26.49 ± 0.39 g). Mice were housed at room temperature (22.0 ± 1.0°C) under a 12-h light/dark cycle. The number of mice per group was set at 24 in accordance with the work of Alhowail and Almogbel [59].

### 1,5-AF administration: SAMP8 mice

Mice were allowed free access to water and a plant-polysaccharide-based chow (Oriental Yeast) supplemented with either 2% 1,5-AF (SUNUS) or 2% erythritol, in accordance with the work of Ito et al. [11]. Body weight, food intake, and water intake were measured at 4-week intervals (intakes did not differ among mice).

### Distribution of mice among experiments

The first phase included 24 mice (12 per group); these mice were used for OF, NOR, immunohistochemical, and immunoblotting analyses. The second phase included 24 mice (12 per group); these mice were used for OF, NOR, and MWM analyses. Mice in the second phase were not used for immunoblotting and immunohistochemical staining because the MWM load can cause molecular changes in brain tissue. In the first phase, behavioral testing began with an OF test; 1 day later, NOR tests were performed. Tissues were collected 1 day after completion of all behavioral tests. In the second phase, behavioral testing began with an OF test; 1 day later, NOR tests were performed; on day 3, MWM analysis was conducted. Tissues were collected 1 day after completion of all behavioral tests.

After excluding mice with abnormal behavior, we analyzed 23 mice in the 1,5-AF group and 22 mice in the control group. The experimental protocol is depicted in Figure 8C.

### OF test

Behavioral and locomotor activity were measured using an OF test when mice were 6 months (1,5-AF: *n* = 23, control: *n* = 22) and 12 months (*n* = 19, *n* = 14) of age. We used the OF test as an indicator of aging-related decline in activity, in accordance with our previous study that validated this approach [9]. Spontaneous activities were recorded for 1 h using a video camera (Logitech HD Pro Webcam C920r) mounted above the OF. Locomotor distance and movement velocity were measured using SMART software, version 3.0 (Panlab, Barcelona, Spain).

### MWM

In the second phase, 12-month-old mice (1,5-AF: *n* = 11, control: *n* = 6) underwent cognitive function assessment using the MWM to measure hippocampal-dependent spatial reference memory.

MWM analysis was performed over 5 days. The MWM comprised a circular swimming pool 120 cm in diameter with a 14-cm-high platform. The pool was filled with water that contained 33 mg of food coloring (11 mg each of red, green, and yellow) to a depth of 15 cm, with a temperature of approximately 22 ± 1°C. The apparatus was marked using external cues in four directions (upper right, upper left, lower right, and lower left). Mice were required to find the platform by following external signs while swimming; they were trained for 4 days before the assessment. On day 5, the platform was removed, and the time that the mouse remained in each quadrant (and on the platform) was recorded. Additionally, the time required for the mouse to find the platform was recorded; if a mouse did not find the platform within 60 s, it was placed on the platform for 10 s. Mouse movements were recorded by a video camera (Logitech HD Pro Webcam C920r) attached to the top of the pool and were analyzed using SMART software, version 3.0 (Panlab).

### NOR test

The NOR test was used to assess recognition memory in 12-month-old mice (1,5-AF: *n* = 19, control: *n* = 14) in accordance with our previous work [9]. The discrimination index was calculated as follows: (time spent exploring novel object – time spent exploring familiar object)/(time spent exploring familiar object + time spent exploring novel object).

### Euthanasia and tissue collection

Animals were euthanized as previously described [50, 51]. Brain tissues were collected from the AIS model rats, SHRSPs, and SAMP8 mice. Tibialis anterior, soleus, and brain tissues were collected from SHRSPs; muscle weights were measured in those rats.

### Measurement of ischemic infarct

Infarct volume measurements were performed as described by Otsuka et al. [56]. Two-mm-thick coronal sections were stained with 1% TTC in phosphate-buffered saline (PBS, pH 7.4) at 37°C for 10 min. Stained sections were scanned with a GT-S640 scanner (Seiko Epson Corporation, Nagano, Japan). Measurements were conducted with ImageJ software, version 1.46r (National Institutes of Health (NIH), Bethesda, MD, USA). Total infarct area (mm³) was multiplied by section thickness to obtain infarct volume.

### Immunoblotting

For the immunoblotting experiments, the cerebral cortex was surgically removed, placed on ice, and homogenized with a tissue protein extraction solution (Thermo Fisher Scientific/Pierce T-PER, 78,510; Thermo Fisher Scientific, Waltham, MA, USA). Protein samples (12 μg each) were separated by electrophoresis in a 4–20% mini-PROTEAN precast gel (Bio-Rad, Hercules, CA, USA), then transferred to a polyvinylidene difluoride (anti-phospho-AMPKα, anti-AMPKα, anti-PGC-1α, anti-FNDC5, anti-BDNF, anti-TNF-α, anti-iNOS, and anti-arginase-1) or nitrocellulose (anti-Iba1) membrane. Membranes were blocked with Polyvinylidene Difluoride Blocking Reagent (NKB101/NYPBR; Toyobo, Osaka, Japan) or 3% skim milk diluted in Tris-buffered saline plus Tween-20 for 1 h at 25°C. Subsequently, each membrane was incubated overnight with primary antibody at 4°C before being incubated with horseradish peroxidase-labeled secondary antibody (goat anti-rabbit IgG H&L (1:4000; Abcam plc, Cambridge, UK) or goat anti-mouse IgG H&L (1:5000; Abcam plc)) at room temperature. Protein bands were visualized via chemiluminescence (WSE-6100 LuminoGraph I; ATTO, Tokyo, Japan) and measured using ImageJ software, version 1.46r (NIH). The following antibodies were used (all diluted 1:1000): rabbit anti-phospho-AMPKα antibody (T172) and rabbit anti-AMPKα antibody (2532S) (both from Cell Signaling Technology, Inc, Danvers, MA, USA), rabbit anti-PGC-1α antibody (ab54481; Abcam plc), rabbit anti-FNDC5 antibody (23995-1-AP; 1:400, Proteintech Group, Inc, Rosemont, IL, USA), rabbit anti-BDNF antibody (BS-4989R; Bioss, Woburn, MA, USA), rabbit anti-TNF-α antibody (ab6671; Abcam plc), rabbit anti-Iba1 antibody (016-20001; Fujifilm Wako Pure Chemicals Co., Osaka, Japan), rabbit anti-iNOS antibody (a marker of M1 microglia; ab15323; Abcam plc), and rabbit anti-arginase-1 antibody (a marker of M2 microglia; 93668; Cell Signaling Technology, Inc). Protein levels were semiquantitatively determined using α-tubulin as an internal loading control.

### Immunohistochemical staining

The brains of all animals were fixed overnight at 4°C with 4% paraformaldehyde in 0.1 M phosphate buffer (pH 7.4). Coronal brain sections (thickness, 4 μm) were then prepared, deparaffinized, and rehydrated. Endogenous peroxidase activity was blocked by incubation with 0.9% hydrogen peroxide in methanol for 10 min at room temperature. Next, the sections were rinsed three times in PBS (pH 7.6) for 5 min each and blocked with 10% skim milk in PBS for 20 min at room temperature. The sections were then incubated with rabbit anti-PGC1-α antibody (ab54481; 1:500, Abcam plc), rabbit anti-FNDC5 antibody (23995-1-AP; Proteintech Group, Inc.), rabbit anti-BDNF antibody (sc-546; 1:100, Santa Cruz Biotechnology, Dallas, TX, USA), and rabbit anti-Iba1 antibody (019-19741; 1:1000, Fujifilm Wako Pure Chemicals Co.) overnight at 4°C. Subsequently, sections were rinsed with PBS and incubated with goat anti-rabbit IgG conjugated to a peroxidase-labeled dextran polymer (EnVision; Agilent Dako, Santa Clara, CA, USA) for 60 min at room temperature. Sections were again rinsed with PBS, and immunoreactivity was visualized by diaminobenzidine staining.

Co-localization of rabbit anti-TNFα antibody (ab6671; 1:100, Abcam plc) and mouse anti-NeuN antibody (ab104224; 1:200, Abcam plc) was examined using immunofluorescence staining. Sections were stained with Alexa Fluor 488-conjugated goat anti-rabbit IgG (A27034; 1:1000; Thermo Fisher Scientific) and Alexa Fluor 555-conjugated goat anti-mouse IgG (A28180; 1:1000; Thermo Fisher Scientific) antibodies for 60 min at room temperature. Next, sections were washed with PBS and mounted in aqueous mounting medium before the immunofluorescence staining was observed under a fluorescence microscope (BZ-X810; Keyence Corporation, Osaka, Japan).

### Quantitative analysis of immunolabeled areas

For the AIS model and SHRSPs, immunostained sections of two areas within the motor cortex were photographed at 20× magnification (0.36 mm^2^ per section) using a microscope and connected camera (DP-21, Olympus Corporation, Tokyo, Japan) (Supplementary Figure 2A, 2B). The mean ratio of immunolabeled area (immunolabeled area/total area) was calculated in the two motor cortex areas. In SAMP8 mice, the left and right hippocampus (CA1 and CA3 regions) and motor cortex were imaged at 20× magnification. The mean ratio of the immunolabeled area (immunolabeled area/total area) was calculated in the left and right areas. Quantitative analyses of immunolabeled areas in the treatment group were conducted by two blinded researchers (Supplementary Figure 2C). The analyses were performed using ImageJ software, version 1.46r (NIH).

### Quantitative analysis of microglial number, area of microglial vesicles, and pericellular area

Iba1-positive microglia were photographed in the same area and magnification as the immunolabeled area images, and the numbers of microglia were counted. Ten Iba1-positive microglia were then randomly selected and their area per cell (μm^2^/cell) and perimeter (μm/cell) were calculated. Quantitative analysis was performed by two blinded investigators using ImageJ software, version 1.46r (NIH).

### Statistical analyses

*P*-values < 0.05 were considered statistically significant. Data are expressed as the mean ± standard error. Statistical analyses were conducted using GraphPad Prism software, version 9.2.0.332 (GraphPad Software, San Diego, CA, USA). In all animals, the log-rank test was used to compare survival rate; the Shapiro–Wilk test was used to confirm normality before parametric or nonparametric tests were used to compare groups. In the AIS model, Student’s *t*-test was used to compare infarct volume, immunoblots (pAMPK, PGC-1, FNDC5, BDNF, TNF-α, Iba1, iNOS, and arginase-1), % of PGC1-α, FNDC5- and BDNF-positive cell areas, numbers of TNF-α- and NeuN-positive cells, and morphological characteristics (number, area, and perimeter) of Iba1-positive cells. The Mann–Whitney *U* test was used to compare neurological scores. In SHRSPs, Student’s *t*-test was used to compare BP, body weight, soleus muscle weight, rotarod fall latency, immunoblots (PGC-1, FNDC5, BDNF, TNF-α, Iba1, iNOS, and arginase-1), % of PGC1-α, FNDC5- and BDNF-positive cell areas, TNF-α- and NeuN-positive cells, and morphological characteristics (number, area, and perimeter) of Iba1-positive cells. The Mann–Whitney *U* test was used to compare tibialis anterior muscle and immunoblots (pAMPK). In SAMP8 mice, mixed models were used to examine the effects of group and time on locomotor activity levels. Moreover, two-way analysis of variance with repeated measures was used to compare the change over time in escape latency to the MWM platform; the Mann–Whitney *U* test was used to compare numbers of platform crossings and recognition index. Student’s *t*-test was used to compare immunoblots (pAMPK, PGC-1, BDNF, TNF-α, Iba1, iNOS, and arginase-1), % of PGC1-α, and BDNF-positive cell areas. The Mann–Whitney *U* test was used to compare FNDC5- and TNF-α-positive cell areas and morphological characteristics (number, area, and perimeter) of Iba1-positive cells.

## Supplementary Materials

Supplementary Figures
