# Correction for: 1,5-anhydro-D-fructose induces anti-aging effects on aging-associated brain diseases by increasing 5’-adenosine monophosphate-activated protein kinase activity via the peroxisome proliferator-activated receptor-γ co-activator-1α/brain-derived neurotrophic factor pathway

**DOI:** 10.18632/aging.206208

**Published:** 2025-02-28

**Authors:** Kiyoshi Kikuchi, Shotaro Otsuka, Seiya Takada, Kazuki Nakanishi, Kentaro Setoyama, Harutoshi Sakakima, Eiichiro Tanaka, Ikuro Maruyama

**Affiliations:** 1 Division of Brain Science, Department of Physiology, Kurume University School of Medicine, Fukuoka 830-0011, Japan; 2 Department of Neurosurgery, Kurume University School of Medicine, Fukuoka 830-0011, Japan; 3 Department of Systems Biology in Thromboregulation, Kagoshima University Graduate School of Medical and Dental Sciences, Kagoshima 890-8520, Japan; 4 Course of Physical Therapy, School of Health Sciences, Faculty of Medicine, Kagoshima University, Kagoshima 890-8544, Japan; 5 Division of Laboratory Animal Resources and Research, Center for Advanced Science Research and Promotion, Kagoshima University, Kagoshima 890-8520, Japan

**Keywords:** AMP-activated protein kinases, brain-derived neurotrophic factor, peroxisome proliferator-activated receptors, blood pressure, aging

**This article has been corrected:** The authors corrected the text of the FUNDING section and the updated text is listed below:

