# Supplementary material for: 1,5-anhydro-D-fructose induces anti-aging effects on aging-associated brain diseases by increasing 5’-adenosine monophosphate-activated protein kinase activity via the peroxisome proliferator-activated receptor-γ co-activator-1α/brain-derived neurotrophic factor pathway
Source: Aging (Albany NY). 2023 Nov 9;15(21):11740–63. doi: 10.18632/aging.205228 (PMC10683599; doi:10.18632/aging.205228)
Supplement: Supplementary Figures [file aging-15-205228-s001.pdf]

SUPPLEMENTARY FIGURES

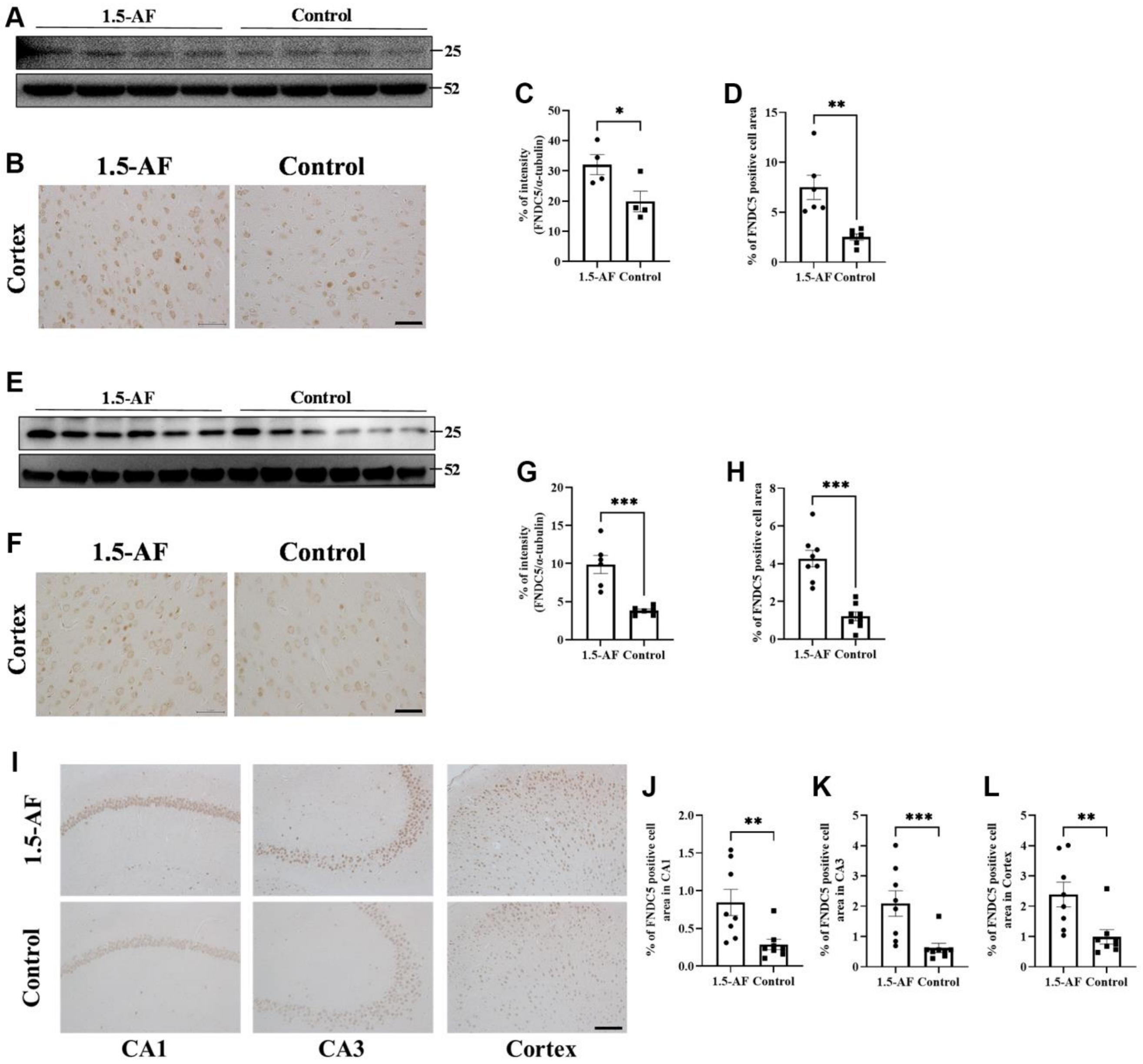

Supplement Figure 1. FNDC5 expression in the brain of AIS, SHRSPs, and SAMP8. Representative immunoblotting of FNDC5 (**A**) and photomicrographs of FNDC5 immunoreactivity (**B**) in AIS. The protein levels of FNDC5 significantly higher in the 1.5-AF rats than in the Control group (**A**, **C**, n = 4) FNDC5 immunoreactivity significantly increased in the 1.5-AF group compared to that in the Control group (**B**, **D**, n = 6). Representative immunoblotting of FNDC5 (**E**) and photomicrographs of FNDC5 immunoreactivity (**F**) in SHRSPs. The protein levels of FNDC5 significantly higher in the 1.5-AF rats than in the Control rats (**E**, **G**, n = 6) FNDC5 immunoreactivity significantly increased in the 1.5-AF rats compared to that in the Control rats (**F**, **H**, n = 8). Representative photomicrographs of FNDC5 immunoreactivity (**I**) in SAMP8. FNDC5 immunoreactivity significantly increased in the 1.5-AF rats compared to that in the Control rats (**J**–**L**, n = 8). Data are shown as the mean± standard error. $^{*}p < 0.05$, $^{**}p < 0.01$, $^{***}p < 0.001$. Scale bar = 50 μm (**B**, **F**) and 100 μm (**I**).

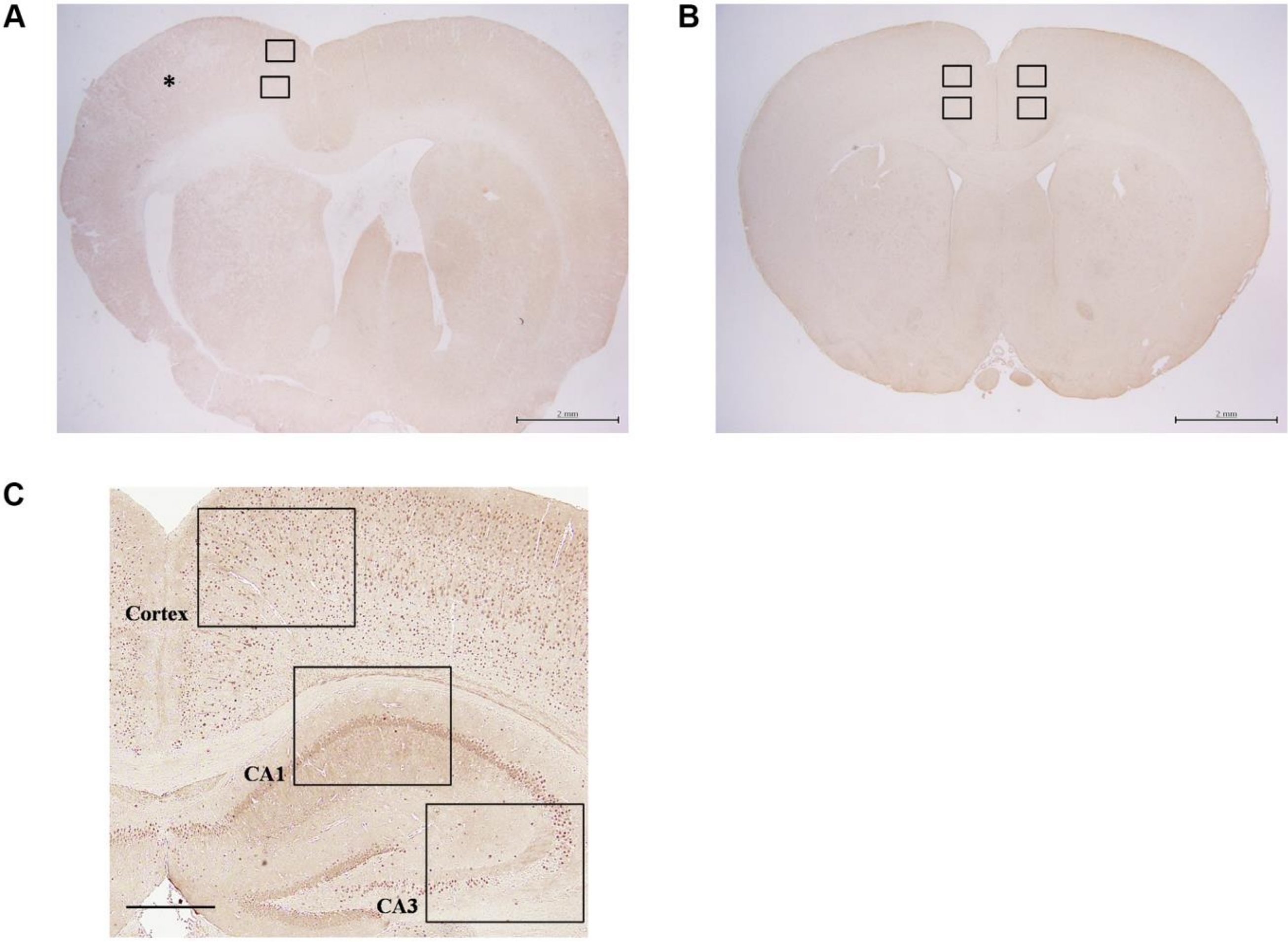

Supplementary Figure 2. Photographs of representative quantitative sites. Photographs of immunostaining in AIS (**A**), SHRSPs (**B**), and SAMP8 (**C**). The area enclosed by the square was quantified. *Penumbra area. Scale bar = 2 mm (**A**, **B**) and 500 µm (**C**).
